# Traditional Uses, Bioactive Compounds, and Pharmacological Investigations of *Calendula arvensis* L.: A Comprehensive Review

**DOI:** 10.1155/2023/2482544

**Published:** 2023-01-03

**Authors:** Aya Khouchlaa, Aicha El Baaboua, Hamza El Moudden, Fatima Lakhdar, Saad Bakrim, Naoual El Menyiy, Omar Belmehdi, Hicham Harhar, Nasreddine El Omari, Abdelaali Balahbib, Moon Nyeo Park, Gokhan Zengin, Bonglee Kim, Abdelhakim Bouyahya

**Affiliations:** ^1^Laboratory of Biochemistry, National Agency of Medicinal and Aromatic Plants, 34025 Taounate, Morocco; ^2^Biology and Health Laboratory, Department of Biology, Faculty of Science, Abdelmalek-Essaadi University, Tetouan, Morocco; ^3^Ecole Supérieure de Technologie d'El Kelaa Des Sraghna, Université Cadi Ayyad, B.P 104, El Kelaa Des Sraghna, Morocco; ^4^Department of Biology, Laboratory of Marine Biotechnology and Environment, Faculty of Sciences, Chouaib Doukkali University, BP 20, El Jadida 24000, Morocco; ^5^Geo-Bio-Environment Engineering and Innovation Laboratory, Molecular Engineering, Biotechnology and Innovation Team, Polydisciplinary Faculty of Taroudant, Ibn Zohr University, Agadir 80000, Morocco; ^6^Laboratory of Pharmacology, National Agency of Medicinal and Aromatic Plants, 34025 Taounate, Morocco; ^7^Laboratory of Nanotechnology, Materials and Environment, Department of Chemistry, Faculty of Science, Mohammed V University in Rabat, Rabat, Morocco; ^8^Laboratory of Histology, Embryology, and Cytogenetic, Faculty of Medicine and Pharmacy, Mohammed V University in Rabat, Rabat 10100, Morocco; ^9^Laboratory of Biodiversity Ecology and Genome, Faculty of Sciences, Mohammed V University, Rabat P.O. Box 1014, Rabat, Morocco; ^10^Department of Pathology, College of Korean Medicine, Kyung Hee University, Seoul 02447, Republic of Korea; ^11^Physiology and Biochemistry Research Laboratory, Department of Biology, Science Faculty, Selcuk University Campus, Konya, Turkey; ^12^Laboratory of Human Pathologies Biology, Department of Biology, Faculty of Sciences, Mohammed V University, Rabat B.P. 1014, Morocco

## Abstract

*Calendula arvensis* L. (Asteraceae) is a famous ornamental and medicinal plant widely distributed in Mediterranean countries and the southern region of Europe. This reputed species is widely used in traditional medicine in the treatment of many disorders and has various bioactivities, especially anti-inflammatory, antiviral, antimutagenic, antimicrobial, insecticidal, antioxidant, and immunomodulatory activities. The present review was conducted to provide a critical review of the comprehensive and current knowledge regarding *C. arvensis* species, in particular, its taxonomy and geographical distribution, botanical description, medicinal uses, phytochemical compounds, pharmacological properties, and toxicity investigations. The data collected on *C. arvensis* were obtained using different scientific research databases such as PubMed, SciFinder, SpringerLink, Web of Science, Science Direct, Google Scholar, Wiley Online, and Scopus. Phytochemical screening of different *C. arvensis* extracts and essential oils showed their richness in bioactive compounds, particularly in fatty acids, sterols, phenolics, flavonoids, saponins, tannins, alkaloids, and terpenoid compounds. The findings of this review showed that the pharmacological activities of *C. arvensis* confirm its importance and diversity as a traditional remedy for many diseases. This plant presents a wide range of bioactivities, namely, anti-inflammatory, antimicrobial, antitrypanosomial, antitumoral, antimutagenic, and immunomodulatory activities, as well as hemolytic properties and wound treatment. Nevertheless, pharmacokinetic validation and toxicological examinations are required to detect any possible toxicity for future clinical trials.

## 1. Introduction

*C. arvensis,* commonly known as “marigold,” is one of the important medicinal herbs belonging to the Calenduleae tribe of the Asteraceae family [1]. It is widely distributed in Mediterranean countries as a native plant [2], usually indigenous to the southern region of Europe, namely Spain, Portugal, Portugal, Turkey, Greece, Malta, and Italy [3], and also cultivated in other parts of the globe such as California and Australia [4]. It is an annual herbaceous plant reaching 100 cm in height [5] and it grows in waste grounds, vineyards, and fields [4]. Its leaves are lance-shaped and have tectorial and secretory trichomes. The inflorescence consists of a single flower head reaching 4 cm with yellow or yellow-orange capitula (blossoms are synthesized throughout the year, but the flowering peaks are between March and July) [6, 7]. Based on the polymorphism of stems, flowers, and achenes, several subspecies were assigned to *C. arvensis* [7], namely, *C. arvensis* var. *parviflora*, *C. arvensis* subsp. *hydruntina*, *C. arvensis* subsp*. macroptera,* and *C. arvensis* subsp. *arvensis* [8]. *C. arvensis* is distinguished from others by the presence of rostrate/bialate [4]. The karyological evidence on *C. arvensis* revealed a 2*n* = 44 and a genome size of 5.41 pg [4, 9]. Phytochemical screening of several essential oils (EOs) and extracts isolated from different parts of *C. arvensis* demonstrated the abundance of bioactive compounds in this plant. These include alkaloids, tannins, saponins, flavonoids, phenolic compounds, sterols, and fatty acids [10–13]. The phytochemical content of *C. avensis* flowers was characterized by a high content of linoleic acid, palmitic acid, and linolenic acid in the extract [10]. The volatile compounds identified from steam distillation extraction of *C. arvensis* were represented by aldehydes, esters, sulfur compounds, alkyl pyrazines, ketones (1-octen-3-one), lactones (*δ*-decalactone), and terpenoid compounds (*α*-terpineol and citronellol) [11]. In traditional herbal medicine, *C. arvensis* was reported to treat many disorders depending on the part used. It can be used as a cataplasm [14] and antidiabetic drug [15, 16], and also against neurological disorders, microbial affections, ENT (Ear, Nose, Throat) diseases, typhoid diseases [17], skin diseases [18], digestive disorders [19], high blood pressure [20], and rheumatic conditions [21], as well as an antispasmodic [22], antiemetic [23], antihelmintic, diaphoretic [18], and antifungal [24] agent.

In Morocco, *C. arvensis* is among the famous plants used in folk medicine, the flowers are the main part used in several regions to treat inflammation and infection in infusion [14, 16]. In addition, flowers and leaves are used as antidiabetic drugs in the northern regions of Morocco [15, 16, 25]. However, in the southern regions, *C. arvensis* is used in traditional medicine to treat neurological, microbial affection, ENT, and typhoid conditions [17], as well as in wound healing [26]. In European countries such as Spain, Italy, and Greece ethnobotanical surveys showed that the aerial part of this plant is used as an emmenagogue, aperitive [27], and ecchymotic [28], and also has an antiseptic effect [29]. Using decoction, the inflorescences have healing [30], emmenagogue, diaphoretic, diuretic, sedative, and anti-inflammatory effects [31]. In Turkey, the aerial part of *C. arvensis* was used as an infusion, mash, lotion, maceration, or oil to treat wounds, burns, and skin cancer, and also for skin care and athlete's foot [32, 33]. The use of leaves and capitulum of this herbaceous plant can be used as a poultice to treat skin diseases. *C. arvensis* flower decoction was used for its hepatoprotective, germicide, and fungal effects [34]. In Asia, *C. arvensis* leaves and flowers in decoction oil form have traditional antirheumatic [21], astringent, and antispasmodic effects [35, 36], with expectorant properties, and also relieve varicose veins [35]. Moreover, these parts have anthelmintic effects and are used as a tonic and diaphoretic agents [37]. *C. arvensis* is an ornamental, medicinal, and industrial plant. Pharmacological investigation of *C. arvensis* leaf EOs and extracts has revealed a broad range of biological properties. Its phytochemical diversity has demonstrated varying levels of antimicrobial effect on various microorganisms. The antimicrobial effect of methanol/chloroform mixture extracts and aqueous extracts of *C. arvensis* leaves was assessed against six bacterial strains (*Enterobacter aerogens*, *Bordetella bronchiseptica*, *Escherichia coli*, *Salmonella typhimurium*, *Staphylococcus aureus*, and *Bacillus subtilis*) and five fungal strains (*Aspergillus niger*, *Muco*r spp., *Aspergillus fumigatus*, *Fusarium solani*, and *Aspergillus flavus*) [38–40]. Moreover, *C. arvensis* extract showed good antibacterial activity against *Mycobacterium smegmatis* with minimal inhibition concentration in the range of 13.2–62.5 *μ*g [41]. Furthermore, *C. arvensis* aerial part EO showed antifungal activity against *Aspergillus niger* and *Penicillium expansum* [38] and did not show any interesting inhibitory effect against yeasts and dermatophyte strains [42]. Regarding the antiparasitic effect, the saponin substances arvensoside B and arvensoside D extracted from *C. arvensis* showed an impressive impact on the parasites of *Trypanosoma brucei* [43]. Additionally, several studies have proven that treatment with *C. arvensis* extracts has excellent anti-inflammatory potential by inhibiting edema formation and managing pain conditions [44, 45]. *C. arvensis* extracts and isolated molecules have also shown effective antioxidant activity in scavenging free radicals, as reported in several investigations [10, 46, 47]. On the other hand, various concentrations of the extracts of *C. arvensis* parts (leaves, stems, and flowers) using different solvents (methanol, ethyl acetate, chloroform, and hexane) recorded potent activities against the proliferation and growth of tumor cells, using a cancer cell model [10, 47, 48]. Enzymatic inhibitory effects of *C. arvensis* leaf and flower extracts have also been revealed and could be related to the presence of compounds derived from triterpenes and flavonoids identified in the different parts of this species [49]. Several other pharmacological activities of *C. arvensis* were also reported. As mentioned above, this medicinal herb could exhibit wound healing and immunomodulatory activities capable of activating lymphocytes and pronouncing blastogenesis and can be considered a potential immune booster [48].

## 2. Research Methodology

The data concerning distribution, botanical description, taxonomy, medicinal use, phytochemistry, and biological properties of *Calendula arvensis* were collected using several scientific search engines, including Wiley Online, SpringerLink, ScienceDirect, Scopus, Web of Science, PubMed, SciFinder, and Google Scholar. The data collected were then organized and classified to be explored and analyzed, and summarized in this work according to each field. For this bibliometric investigation, different keywords linked to *Calendula arvensis* were used, including *Calendula arvensis*, *Calendula arvensis* essential oils, *Calendula arvensis* extracts, and biological effects of *Calendula arvensis*. Regarding the phytochemical data, the IUPAC names of the identified phytochemicals were verified using the PubChem database. Bioactive compounds were designed using ChemDraw Pro 8.0 software.

## 3. Results and Discussion

### 3.1. Botanical Description

**Figure 1:**
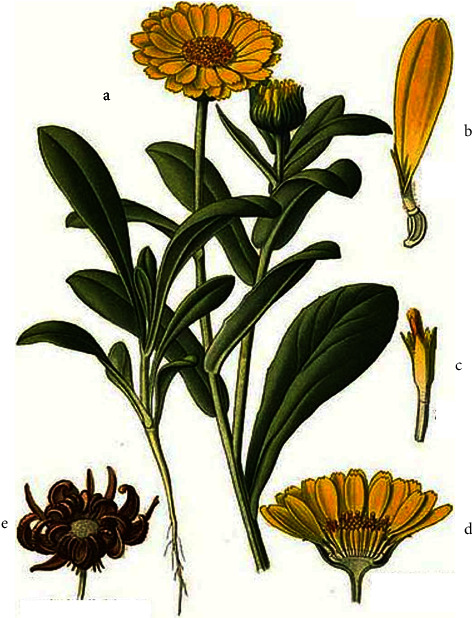
*C. arvensis* (a) flowering branch; (b) ray floret; (c) tube flowers; (d) flower head; (e) achene flower head.

**Figure 2:**
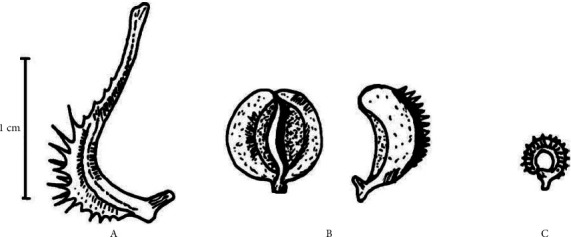
Morphology of *C. arvensis* achenes: (a) rostrate; (b) cymbiform; (c) annular.

*Calendula arvensis*, as depicted in Figure 1, is an annual herb that can reach 100 cm in height [5]. The leaves are lance-shaped and possess secretory and tector trichomes. The inflorescence consists of a single flower head up to 4 cm with yellow or yellow-orange capitula (blossoms appear throughout the year, but flowering peaks between March and July) [6, 7]. The peripheral flowers of the capitula (ligulate) extend over several rows (verticils) and are female; central flowers (tubular) are complete but functionally male [6]. The achenes are only produced by the ligulate flowers. The pollen is echinate, tetracolporate and monadic. The shape of pollen in the equatorial view is circular to perprolate and the polar view is rectangular [50]. Thus, a complex polymorphism is related to the position of the flowers in the capitula, with three achene types (cymbiform, rostrate, and annula) (Figure 2), with a stem size of 5–60 cm and a leaf width of 5–20 mm [6]. Due to stem, flower, and achene polymorphism, different subspecies were assigned to *C. arvensis* [7], viz., *C. arvensis* var. *parviflora*, *C. arvensis* subsp. *hydruntina*, *C. arvensis* subsp. *macroptera*, and *C. arvensis* subsp. *arvensis* [8]. The karyological data on *C. arvensis* showed a 2*n* = 44 and a genome size of 5.41 pg [4, 9]. *C. arvensis* is differencied from others by the presence of bialate/rostrate [4].

### 3.2. Taxonomy and Geographic Distribution

*C. arvensis* belongs to the Calenduleae tribe of the Asteraceae family which includes about 25 annual and perennial species [1]. The genus *Calendula* consists of about 15 species in the Mediterranean, Saharo-Arabian, and Irano-Turanian regions [51], native to the Mediterranean countries [2] from Macaronesia to Southeast Asia [4], and generally native to the southern region of Europe, including Spain, Portugal, Turkey, Greece, Malta, and Italy [3]. The genus *Calendula* belongs to other parts of the globe such as California and Australia [4]. *C. arvensis*, a field marigold, is abundant in spring, annually, and weeds. It is found gregarious in field margins, graveyards, roadsides, wastelands, and open places [47]. It grows in waste grounds, vineyards, and fields [4].

### 3.3. Ethnomedicinal Use

Several ethnobotanical studies have marked the importance of *C. arvensis* in traditional medicine to treat many disorders. The traditional use of *C. arvensis* is related to the part used. Indeed, different parts of *C. arvensis* have traditionally been used to treat different diseases.  Table 1 lists the application of *C. arvensis* in traditional pharmacopeia global systems.

The flowers represent the main used part in Moroccan traditional systems in several regions to heal various disorders. The population of the Targuist region (Northern Morocco) used the flowers of *C. arvensis* as a cataplasm against inflammation and infections [14]. The population of Rabat-Sale-Kenitra regions (Morocco) used flowers and leaves as an antidiabetic drug [15]. Also, the same use of this plant against diabetes was reported by an ethnobotanical study conducted in the Rif region (North of Morocco) [16] and in the Taza region (North-East of Morocco) [25]. Chaachouay et al. [16] reported the use of the flowers of *C. arvensis* in infusion, while Naceiri Mrabti et al. [25] reported the use of flowers and stems of *C. arvensis* in decoction. In the northwest of Morocco, the population of Oulad Daoud Zkhanine (Nador Province) used the decoction of whole plants against digestive disorders as well as for hair care [19]. Other ethnobotanical surveys, conducted in the Aguelmouss region (Khenifra Province, Morocco), have reported the importance of *C. arvensis* in traditional medicine, without mentioning the part used and the mode of preparation, in healing [26], and in the treatment of neurological and microbial conditions, and diseases related to ENT and typhoid [17]. These last two sections are important knowledge in the database ethnobotanical studies, which directs researchers to target a specific part in the purpose to elaborate the phytochemical screening. Thus, a standard form of ethnobotanical surveys is primordial to conserve traditional herbal remedies.

In Western Algeria, *C. arvensis* flowers in infusion were used for depurative, emmenagogue, and antispasmodic effects as well as for stimulating hepatic activity and bile secretion. By maceration, the leaves were used to treat internal ulcers, warts, fistulas, frostbite, calluses body, and skin lesions and to calm vomiting [60]. The flowers of the courant plant were used by the M'Zab Valley population (Algeria) to treat high blood pressure [20]. Another ethnobotanical survey in the Bissa region (north-east of the Dahra mountains in Algeria) indicated the therapeutic effects of *C. arvensis* leaves and roots on rheumatism [74]. In the mountains of Tlemcen (Western Algeria), this plant has been reported to exert disinfectant, anti-influenza, antitranspiration, and antihemorrhagic effects, as well as to treat hemostatic and hepatic actions [63], thus this plant is traditionally known in the Tiaret mountains (Western Algeria) as a depurative, emmenagogues, and antispasmodic [59].

The population of Alaşehir (Manisan, Turkey) and Sarigöl district (Manisa, Turkey) used the aerial part of *C. arvensis* as an infusion, mash, lotion, maceration, or oil to treat wounds, burns, skin cancer, and also for skin care and athlete's foot [32, 33]. Uzun and Kaya [76] confirmed the use of leaves and capitulum of this plant by the population of Mihalgazi (Eskişehir, Turkey) as a poultice to treat skin diseases. The decoction of *C. arvensis* flowers was used by the Erzincan population (Turkey) as a germicidal, hepatoprotective, and fungal agent [34]. A recent ethnobotanical study conducted by Aslan et al. [24] reported the use of *C. arvensis* flower decoction as an antifungal by the population of Yaslıca town and Arıkök neighborhood in the Bozova district of Şanlıurfa province (Turkey). In Syria's western region (Tartus and Latakia), the aerial part of *C. arvensis* was used to treat varicose veins, sore eyes, wounds, sprains, stings, bites, and skin problems [72].

In European countries, several authors have reported the traditional use of *C. arvensis* to treat different illnesses. In Spain, *C. arvensis* aerial part was used as an emmenagogue and aperitive by the population of the Canary Islands [27]. In the same country, the inflorescence (in the form of a liniment) was effective against ecchymotic [28]. Gras et al. [29] showed that the aerial part of the *C. arvensis* bath exhibited an ocular antiseptic effect. As a poultice or infusion, the flowers of this plant were used in the villages of Lotzorai and Escolca (Sardinia, Italy) as antispasmodic and to treat burns [22]. In decoction, the inflorescences have a healing effect [30] as well as an emmenagogue, diaphoretic, diuretic, sedative, and anti-inflammatory effects [31]. In 2016, the population of the Mainarde Mountains (central-southern Apennine, Italy) used *C. arvensis* after maceration or as an ointment against burns, sunburn, chilblains, joint pain, and stings [62]. The flowers of *C. arvensis* have been used by the population of Bosnia and Herzegovina against lung and liver cancer, and skin disorders [73]. Another ethnobotanical study conducted in Greece, [61] showed that the flowers, stems, and leaves of *C. arvensis* were used to treat swollen areas, acne, and diaper rash.

In Pakistan, the traditional use of *C. arvensis* was confirmed by several ethnobotanical studies (Table 1). As a poultice, this species was consumed by the population of Tehsil Barawal, Upper Dir, Khyber Pakhtunkhwa (Pakistan) as a tonic and inflammatory agent against eye troubles and oral sores [53]. The flowers of *C. arvensis* were used by the population of Attock district (Pakistan) to strengthen eyesight, heart diseases and to heal the skin [55]. The same effects were confirmed by Rehman et al. [52] and Jan et al. [69] in the Khattak tribe population of Chonthra Karak (Pakistan) and by the Kohistan Valley population, Khyber Pakhtoonkhwa (Pakistan). Another ethnobotanical study of this plant in the tribal communities of North-West Frontier Province (Pakistan) indicated that the leaves of *C. arvensis* were used for wound healing [36, 54]. Khan et al. [70] confirmed the use of *C. arvensis* as a wound healer by the population of the Sawans Valley Mianwali, Punjab (Pakistan). This plant's flowers and leaves were used as stimulant, antispasmodic, astringent, healing wounds and injuries [67], as well as in the treatment of scrofula, diaphoretic, anthelmintic, discharge of mucus, and helminthic [64–66]. Other ethnobotanical studies showed that *C. arvensis* leaves and flowers in decoction oil form have traditional effects as antirheumatic (Tehsil Razzar District Swabi, Pakistan) [21], astringent, and antispasmodic [35, 36], with expectorant properties, and also to relieve varicose veins [35]. Ali et al. [37] showed that the Malam Jabba Valley population of Swat (Pakistan) used the leaves and flowers of *C. arvensis* as an anthelmintic, tonic, and diaphoretic agents. Other ethnobotanical studies at Madyan Valley in district Swat (Pakistan) Malam Jabba, Swat (Pakistan), the Jatlan Azad Jammu, Kashmir region (Pakistan), and the Azad Jammu And Kashmir state (Pakistan) confirmed the anthelmintic, tonic, and diaphoretic actions of *C. arvensis* [18, 23, 56, 57]. Leaves and flowers of *C. arvensis* have been known to be used in traditional pharmacopeia by the population of the Karak, Talash Valley of Lower Dir (Pakistan) and Khyber Pakhtunkhwa district (Pakistan) against toothache [58, 71, 78]. The leaves and shoots of *C. arvensis* were used by the population of Chagharzai Valley, district Buner (Pakistan) against skin diseases [75]. Recently, this plant's stems and leaves were used against diabetes in the South-West of Pakistan [77]. In 2020, the population of the Gujranwala region, Punjab (Pakistan) used the leaves and flowers as extract preparation mode to treat severe pains [68].

### 3.4. Phytochemistry

Medicinal plants secrete different chemical compounds as secondary metabolites, including terpenoids, flavonoids, phenolic acids, alkaloids, and others. These bioactive compounds exhibited several biotactical activities such as antimicrobial, anticancer, antidiabetic, anti-inflammatory, and antioxidant effects [79–83].

**Figure 3:**
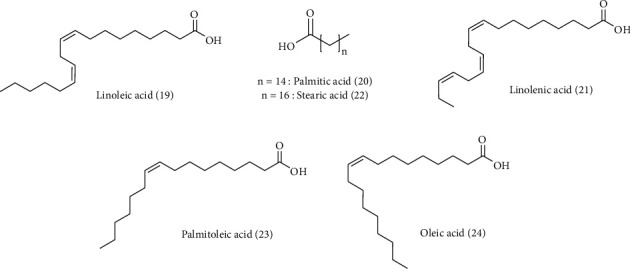
Structure of fatty acid compounds detected in *C. arvensis.*

**Figure 4:**
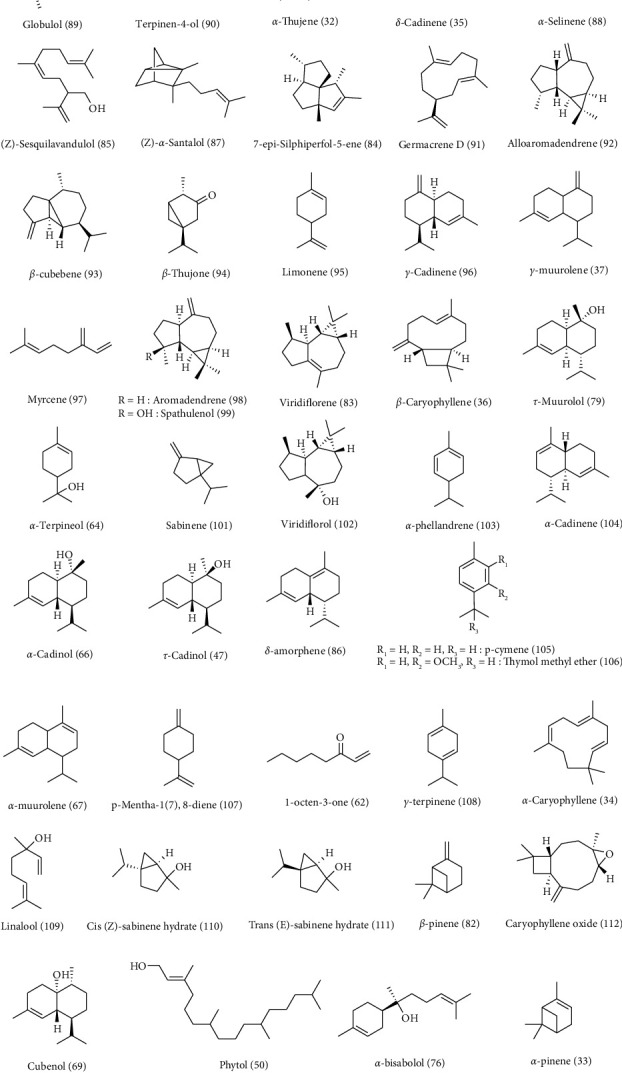
Structure of terpenoid compounds detected in *C. arvensis.*

**Figure 5:**
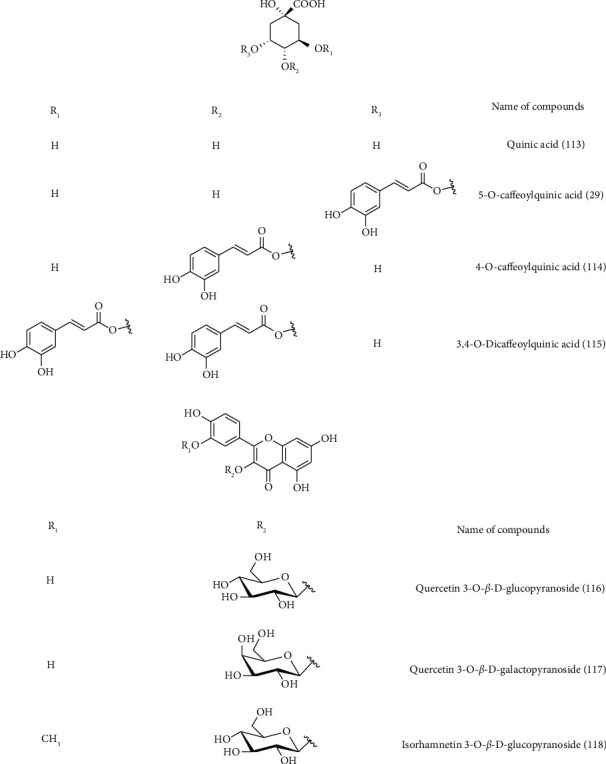
Structure of phenolic compounds detected in *C. arvensis.*

**Figure 6:**
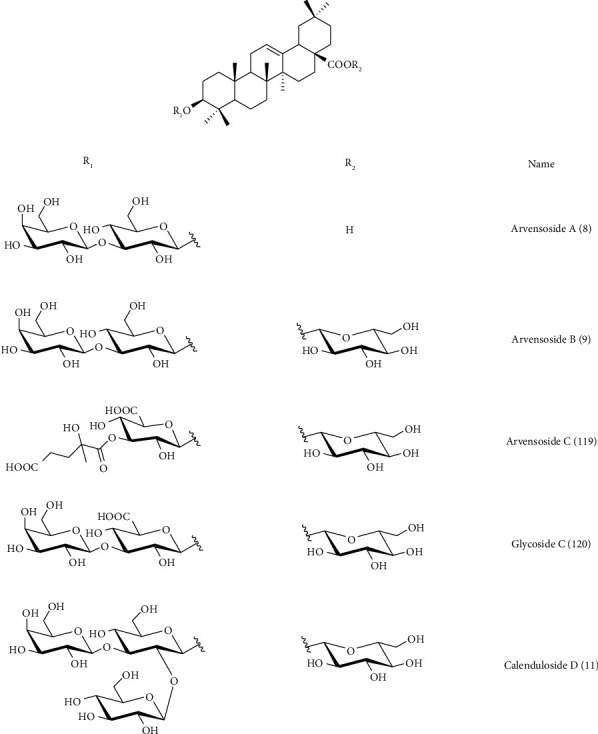
Structure of saponin compounds detected in *C. arvensis.*

**Figure 7:**
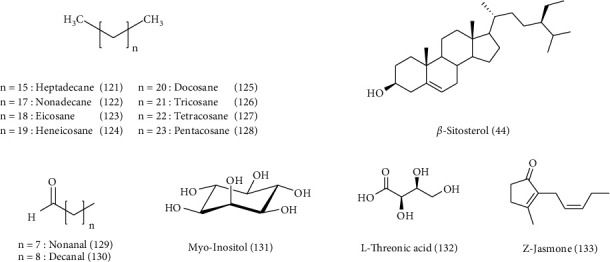
Structure of other compounds detected in *C. arvensis.*

*C. arvensis* secondary metabolites were the subject of multiple works. Almost the majority of them have been on the aerial parts. Phytochemical screening of different *C. arvensis* extracts and essential oils showed their richness in biomolecules. These are most often fatty acids, sterols, phenolics, flavonoids, saponins, tannins, alkaloids, and terpenoid compounds (Table 2 and Figures 3–7).

Pizza and de Tommasi [84] isolated and determined the structure of four triterpenoid saponins from methanolic extract of *C. arvensis* aerial parts, these four compounds are 3-O-(*β*-D-galactopyranosyl-(1⟶3)-*β*-D-glucopyranosyl) oleanolic acid-28-O-*β*-D-glucopyranoside**(1)** (98 mg/2.2 g of MeOH extract); 3*β*-O-(*β*-D-galactopyranosyl-(1⟶3)-*β*-D-glucopyranosyl) oleanolic acid **(2)** (30 mg/2.2 g of MeOH extract); 3*β*-O-(*β*-D-galactopyranosyl-(1⟶3)-*β*-D-glucopyranosyluronic acid) oleanolic acid-28-O- *β*-D-glucopyranoside**(3)** (15 mg/2.2 g of MeOH extract) and 3*β*-O-(*β*-D-galactopyranosyl-(1⟶3)-*β*-D-glucopyranosyluronic acid) oleanolic acid **(4)** (10 mg/2.2 g of MeOH extract). A similar study was elaborated on the same saponin separation pathway in order to identify four compounds 4-O-(*β*-D-fucopyranosyl)-4-alloaromadendrole **(5)**, and three derivatives of arvoside B. In addition, De Tommasi et al. [87] have isolated the same compounds and two other saponins which are 3-O-{*β*-D-ga1actopyranosyl-(1⟶3)(*β*-D-glucopyranosyl-(1⟶4))-*β*-D-glucopyranosyl}oleanolic acid (28⟶1)-*β*-D-glucopyranosyl ester **(6)** and 3-O-{*β*-D-ga1actopyranosyl-(1⟶3)(*β*-D-glucopyranosyl-(1⟶4))-*β*-D-glucopyranosyl}oleanolic acid **(7)** from the aerial parts of *C. arvensi* [87].

On the other hand, two new triterpenoid glycosides from *C. arvensis* aerial parts were identified as arvensoside A **(8)** and B **(9)** by FAB, FAB-MIKE mass spectrometry, and ^13^C NMR spectroscopy.

The study by Vidal-Ollivier et al. on *C. arvensis* was based on the chemical and physicochemical evidence to isolate four compounds, **(8)**, **(9)**, calenduloside C **(10),** and D **(11)** from fresh-aerial parts of *C. arvensis*, two saponosides of these four compounds showed anti-inflammatory activities.

De Tommasi et al. [87] reported several compounds, after analysis of chloroform extracts of *C. arvensis* aerial parts, compounds **(1), (2), (3), (4),** and 4-O-(*β*-D-fucopyranosyl)-4-alloaromadendrole **(12)**, the two new alloaromadendrolglycosides were identified, such as 4-O-(*β*-D-fucopyranosyl)-4-alloaromadendrol-2″-methylpropanoyl esters **(13)** and 4-O-(*β*-D-fucopyranosyl)-4-alloaromadendrol-2″-methyl-2″-butenoyl esters **(14)**.

The structures of the new substances were established by high-field^1^H-NMR spectroscopy on the aerial part extracts of Italian *C. arvensis* which provided four new sesquiterpene glycosides, namely, 3*α*,7*β*-dihydroxy-5*β*,6*β*-epoxyeudesm-4(15)-ene-11-(O-*β*-D-fucopyranoside-2′,4′-diangelate-3′-acetate) **(15)**; 3*α*,7*β*-dihydroxy-5*β*,6*β*-epoxyeudesm-4(15)-ene-11-(O-*β*-D-fucopyranoside-2′,4′-diangelate-3′-isobutyrate) **(16)**; 3*α*,7*β*-dihydroxy-5*β*,6*β*-epoxyeudesm-4(15)-ene-11-(O-*β*-D-fucopyranoside-2′,4′-diangelate-3′-methylbutyrate) **(17),** and 3*α*,7*β*-dihydroxy-15-acetoxyeudesm-4(5)-ene-11-(O-*β*-D-fucopyranoside-2′,4′-diangelate-3′-acetate) **(18)**, in addition to three known compounds [12].

Phytochemical determination of the aqueous and methanolic extracts detected their richness in phenolics and flavonoids. The total phenolic contents ranged from 47.89 ± 2.34 to 50.26 ± 0.18 mg GAE/g DW for the aqueous and methanolic extracts, respectively. The total flavonoid contents were 74.93 ± 1.50 and 174.93 ± 5.21 mg RE/g DW for the aqueous and methanolic extracts, respectively [47].

Phytochemical analyses of *C. arvensis* flower ethyl acetate extract identified phenols (354 *μ*g/mL) and flavonoids (270 *μ*g/mL), and the GC-MS analysis revealed a high content of linoleic acid **(19)** (14.12%), palmitic acid **(20)** (20.28%), and linolenic acid **(21)** (59.12%) in the extract. Stearic **(22)**, palmitoleic **(23)**, and oleic **(24)** acids were present in small amounts [10].

The phytochemical content of *C. avensis* leaves from Pakistan was studied by Akhtar et al. [49]. Qualitative analysis of phytochemicals showed that this plant contains several secondary metabolites such as alkaloids, saponins, tannins, and terpenoids. For the quantitative analysis of phytochemicals, the total phenolic content of the samples ranged from 20.2 to 85.6 mg/g DW in methanol/chloroform extracts and from 5.5 to 62.1 mg GAE/g DW in aqueous extracts. The total flavonoid content ranged from 2.9 to 44.5 mg QE/g DW of the sample for the methanol/chloroform extracts and from 2.4 to 37.1 mg QE/g DW for the aqueous extracts [38]. The *C. arvensis* flower methanol extract was the richest in flavonoids (74.14 ± 3.09 mg QE/g extract) and phenolics (118.18 ± 10.29 mg GAE/g extract) [49].

Phytochemical screening of all extracts (distilled water, 50% aqueous/ethanol, ethanol, chloroform, and petroleum ether) was carried out and showed the presence of proteins, alkaloids, terpenoids, and flavonoids. Petroleum ether extract has been shown to be rich in flavonoids, and the ethanolic extract contained alkaloids, flavonoids, and proteins. Additionally, two phytochemical groups' flavonoids and terpenoids were revealed in the aqueous/ethanolic extract, and the chloroform extract showed the presence of terpenoids, alkaloids, and proteins. Finally, these substances were not identified in the aqueous extract [48].

Four saponins were isolated from the dried aerial parts of *C. arvensis* by Elias and Meo [89] and the identification was carried out by mass spectrometry, FAB, ^1^H- and ^13^C-NMR, and two-dimensional NMR studies. These compounds were **(2)**, **(3)**; 3-O-(*β*-D-glucopyranosyl-(1⟶2)- *β*-D-galactopyranosyl-(1⟶3)-*β*-D-glucopyranosyl) oleanolic acid **(25)**; and 3-O-(*β*-D-glucopyranosyl-(1⟶2)- *β*-D-galactopyranosyl-(1⟶3)-*β*-D-glucopyranosyl) oleanolic acid-28-O- *β*-D-glucopyranoside**(26)** [89].

*C. arvensis* hexane extracts were analyzed by GC–MS allowing the identification of 34 compounds, namely a 1,2-saturated pyrrolizidine alkaloid derivative **(27)** (0.57 mg/g of the dry plant), sesquiterpene 8,14-cedranoxide **(28)** (0.70 mg/g of the dry plant), and **(21)** (0.78 mg/g of the dry plant) the only ones detected in this species were the three main compounds of their hexane extract. However, carbohydrates were the most abundant, together accounting for approximately 30.7%, followed by terpenoids and carboxylic acids with 27.9% and 27.4%, respectively [42].

Overall, UPLC-MS analysis identified 26 compounds in the studied methanolic extract of *C. arvensis*. This extract is clearly enriched with quercetin derivatives and caffeic acid. In the methanolic extract, saponins and hydroxybenzoic acids were also determined. Quantitatively, *C. arvensis* methanol extract was dominated by flavonoids (53.5%) while hydroxycinnamic and hydroxybenzoic acids shared approximately 20%. In this extract, the main compounds were the 5-O-caffeoylquinic acid **(29)** (0.221 ± 0.016 mg/100 mg extract), protocatechuic acid pentoside **(30)** (0.366 ± 0.073 mg/100 mg extract), and quercetin **(31)** (0.534 ± 0.042 mg/100 mg extract) [42].

Sixty-six constituents were detected in *C. arvensis* flower extract. Volatile compounds have been subdivided into seven chemical classes, namely terpenes (36 compounds), sesquiterpenes (32 compounds), ketones (4 compounds), esters (25 compounds), aliphatic hydrocarbons (4 compounds), aldehydes (8 compounds), and alcohols (7 compounds). The most abundant terpene compounds in *C. arvensis* were *α*-thujene**(32)** (788 *μ*g/100 g flower) followed by *α*-pinene**(33)** (268 *μ*g/100 g flower). Regarding the number of compounds, sesquiterpenes were the second most abundant chemical class in this plant, with 28 constituents identified. However, seven of them have only been tentatively determined as sesquiterpene-like molecules. the main sesquiterpene was *α*-caryophyllene**(34)** (118 *μ*g/100 g flower), followed by *δ*-cadinene**(35)** (31.5 *μ*g/100 g flower) and *β*-caryophyllene**(36)** (26.4 *μ*g/100 g flower) and finally *γ*-muurolene**(37)** (22.6 *μ*g/100 g flower) [91]. Nevertheless, *C. arvensis* water/acetone extract exhibited the lowest amount of all the bioactive compounds studied, with total flavonoids of 0.35 ± 0.10 mg QE/g DW, hydrolyzable tannin of 3.7 ± 1.8 mg TAE/g DW, and total monomeric anthocyanin of 0.07 ± 0.01 mg Cy 3-glu/g DW [91].

The total contents of phenolic and flavonoid compounds in the methanolic extracts of *C. arvensis* were, respectively, 14.49 + 0.38 and 5.26 + 0.36 mg/g and in the aqueous extract of 15.12 + 0.40 and 5.31 + 0.36 mg/g [92]. The total content of phenolic compounds in *C. arvensis* methanolic and aqueous extracts was 12.3 and 16.8 mg GAE/g DW, respectively [97].

Furthermore, from the aerial parts of *C. arvensis* (butanolic and ethyl acetate extracts), a novel triterpene saponin, called arvensoside C **(38)**, has been isolated along with four other known **(8)**, **(9)**, **(11)**, and glycoside C **(39)**. Three known flavonol glycosides, namely quercetin 3-O-*β*-D-galactopyranoside**(40)**, quercetin3-O-*β*-D-glucopyranoside**(41)**, and isorhamnetin 3-O-*β*-D-glucopyranoside**(42)** have also been characterized. Their structures have been elucidated by 1D and 2D NMR experiments, including HSQC, DQF-COZY, 1D-TOCSY, and HMBC spectroscopy [93]. Similar studies were reported on the isolation and purification of **(9)** and arvensoside D **(43)** [43]; [95]. GC-MS analysis of chloroform extracts of *C. arvensis* samples from Portugal showed that the most abundant compound was *β*-sitosterol**(44)** [94]. The phytochemical composition of Algerian *C. arvensis* aerial part EOs and hydrosol extract was studied by GC/MS and GC-FID. However, the hydrosol extract and the essential oil were rich in sesquiterpene compounds (81.8%) in which the oxygenated compounds were superior to hydrocarbons with 53.4% and 28.4%, respectively. The major compounds were *γ*-curcumene**(45)** (5.4%), (E,Z)-farnesol **(46)** (6.5%), *τ*-cadinol**(47)** (7.5%), zingiberenol 2 **(48)** (8.3%), eremoligenol **(49)** (9.5%), (E)-phytol **(50)** (10.8%), *β*-curcumene**(51)** (12.5%), and zingiberenol 1 **(52)** (19.1%) [88].

Volatile compounds from the steam distillation extraction of *C. arvensis* were analyzed by GC/MS. The compounds detected were represented by aldehydes ((E,E)-2,4-octadienal **(53)**, (E)-2-nonenal **(54))** and 5-methyl-2-furanaldehyde **(55)**, esters (ethyl butyrate **(56)**, ethyl hexanoate **(57))**, phenethyl acetate **(58)**, sulfur compounds (2-methyl-3-furanthiol **(59)** and methional **(60)**), alkyl pyrazines (2,6-dimethyl-3-ethyl pyrazine **(61)**), ketones (1-octen-3-one **(62)**), lactones (*δ*-decalactone**(63)**), and terpenoid compounds (*α*-terpineol**(64)** and citronellol **(65)**) [11]. Paolini et al. [7] elucidated the *C. arvensis* EO chemical composition by the combination of GC/MS and GC. Consequently, this oil had a high content of sesquiterpenes (87.3 g/100 g of oil) containing a high variety of phytochemical classes in low concentrations. The major sesquiterpenes were *α*-cadinol**(66)** (12.4 g/100 g of oil) and compound **(35)** (15.1 g/100 g of oil). The minor compounds identified were *α*-muurolene**(67)** (4.9 g/100 g of oil), cubebol **(68)** (3.7 g/100 g of oil), cubenol **(69)** (3.5 g/100 g of oil), cubeban-11-ol **(70)** (3.2 g/100 g of oil), germacradien-11-ol **(71)** (3.2 g/100 g of oil), 1-epi-cubenol**(72)** (3.8 g/100 g of oil), 4-epi-cubebol**(73)** (4.5 g/100 g of oil), guaiol **(74)** (1.5 g/100 g of oil), ledol **(75)** (1.9 g/100 g of oil), *α*-bisabolol**(76)** (1.7 g/100 g of oil), calamenene (cis and/or trans) **(77)** (4.7 g/100 g of oil), presilphiperfolane-9*α*-ol**(78)** (4.6 g/100 g of oil), *τ*-muurolol**(79)** (1.2 g/100 g of oil), eremophila-1(10),7-diene **(80)** (1.9 g/100 g of oil), and compound **(37)** (0.1 g/100 g of oil) [7].

Servi et al. [96] also determined the EO components of the *C. arvensis* aerial part by GC-MS analyses. Thirty-six components were identified. The major compounds were ledene (81) (5.1%), (72) (5.4%), (68) (7.2%), (69) (7.7%), (66) (8.5%), (72) (10.7%), and (35) (14.8%) compounds [96].

The GC-MS and GC-FID analysis of *C. arvensis* EO extracted by microwave distillation (MD) and hydrodistillation (HD) revealed a total of 44 and 45 components, representing more than 84.8% and 88.3% of the oil composition, respectively. Monoterpene compounds (HD: 26.3% and MD: 24.3%) and sesquiterpenes (HD: 30.5% and MD: 23.4%) were found to be the major group of volatile compounds. The main terpene compounds of *C. arvensis* EOs were *β*-pinene (82) (HD, 1.8% and MD, 2.4%), viridiflorene (83) (HD, 2.5% and MD, 1.7%), 7-epi-silphiperfol-5-ene (84) (HD, 2.6% and MD, 3.7%), (Z)-sesquilavandulol (85) (HD, 4.8% and MD, 0.0%), *δ*-amorphene (86) (HD, 0.0% and MD, 8.0%), (Z)-*α*-santalol (87) (HD, 8.2% and MD, 7.4%), (33) (HD, 11.9% and MD, 12.3%), and *α*-selinene (88) (HD, 16.0% and MD, 0.0%) [41].

### 3.5. Pharmacological Properties

**Figure 8:**
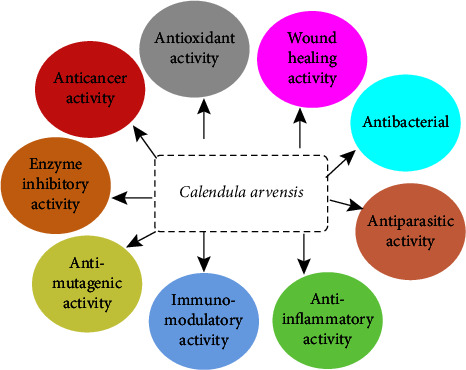
Biological properties of *C. arvensis.*

*Calendula arvensis* is an important medicinal, industrial, and ornamental plant with different bioactivities described in the bibliography, especially as anti-inflammatory [44, 45], antiviral [87, 98], insecticidal [99], antitrypanosomial [43], anticholinesterase [49], antimutagenic [89], immunomodulator [48], hemolytic [100], and wound healing [101] agent (Figure 8).

#### 3.5.1. Antimicrobial Activity

*C. arvensis* is one of the most interesting medicinal plants, its diverse chemical composition displays a varying degree of antimicrobial activity on different microorganisms. Jamal et al. [40] showed that *C. arvensis* leaf extract exhibits an important inhibition on some microorganisms including pathogens using the agar diffusion method and the tube dilution method to define the minimum inhibitory concentration (MIC). The study showed that the potential of *C. arvensis* as a source of antibacterial agents can be utilized in the healthcare delivery process. Indeed, *C. arvensis* chloroform and petroleum ether extract exhibited, respectively, an inhibition at MIC = 2 *μ*g/mL against *Klebsiella pneumoniae* and *Escherichia coli*. The greatest inhibition zone is produced by the chloroform extract at 512 mg/mL against *E. coli*. *C. arvensis* leaf extract was found to have better antibacterial activity in petroleum ether with a zone of inhibition of 1.9 cm.

Likewise, the study of Jamal et al. [40] showed that the organic extracts of *C. arvensis* flowers have an important antibacterial potency evaluated by the agar well diffusion method and by the microtitration technique. Except for *Acinetobacter baumannii*, *Proteus mirabilis*, and *Listeria monocytogenes*, the methanol extract of *C. arvensis* flowers had remarkable activity against the bacteria studied (Gram-positive and Gram-negative). *C. arvensis* flower hexanolic extract inhibited all bacteria except *Acinetobacter baumannii*, *Streptococcus agalactiae*, and *Staphylococcus aureus* MRSA. The MIC value of the methanol extract was between 12.5 and 25 *μ*g/mL and the MIC value of the hexanolic extract was between 6.25 and 12.5 *μ*g/mL. Therefore, the hexane extract was more potent than the methanol extract. The hexanolic extracts of *C. arvensis* flowers obtained by soxhlet extraction were bacteriostatic for all the bacteria studied (*E. coli* MDR, *E. coli*, *E. coli* ATCC, *E. coli enteropathogenes*, *Salmonella braenderup*, *Salmonella aequatoria*, and *Salmonella blockley*), while aqueous and methanolic extracts obtained by maceration in cold water showed bactericidal activity. Moreover, the extracts studied did not show any activity on *Candida* species with the exception of the methanolic extract, which inhibited *Candida famata* and *Candida tropicalis* with inhibition diameters of 20 and 14 mm, respectively [47]. Zoufan et al. [39] showed the antibacterial effect of ethanolic and methanolic extracts of whole *C. arvensis* examined by the disc diffusion method is greatest against *P. aeruginosa*, *S. aureus,* and *E. coli*.

However, the antimicrobial effect of chloroform/methanol mixture extracts and aqueous extracts of *C. arvensis* leaves, assessed against six bacterial (*Escherichia coli*, *Salmonella typhimurium*, *Staphylococcus aureus*, *Bacillus subtilis*, *Bordetella bronchiseptica*, and *Enterobacter aerogens*) and five fungal strains (*Aspergillus niger*, *Muco*r spp., *Aspergillus fumigatus*, *Fusarium solani*, and *Aspergillus flavus*) using the disc diffusion method, showed no activity against the different strains tested [38].

The antifungal activities of *C. arvensis* aerial part EOs and hydrosol extract were investigated against two phytopathogenic fungi; *Penicillium expansum* and *Aspergillus niger* using the agar well diffusion method. The obtained results *in vitro* showed that *C. arvensis* aerial part EO has an antifungal activity at high concentrations, while the hydrosol extract induced the best inhibition against *A. niger* and *P. expansum*. The essential oil (60 mg/L) completely inhibited the growth of *P. expansum* with an important inhibitory activity against *A. niger* (86.6%). At a concentration of 30 mg/L, the hydrosol extract exerted a strong inhibition (100%) against both fungi.

Furthermore, the treatments of pears with *C. arvensis* L. aerial part hydrosol extract and EO showed a very marked protective activity on the severity of the infection caused by *P. expansum*. Against rot induced by *P. expansum*, the hydrosol extract (0.02 mg/L) showed a high protective effect (100%) for up to 7 days. At the highest EO concentration (0.2 mg/L), protective effects of 100% and 40% were observed up to the 7^th^ and 9^th^ day, respectively, and this was sufficient to reduce the disease severity [88]. According to Faustino et al. [42], methanol extracts of the *C. arvensis* sample did not show any interesting antimicrobial activity against *Aspergillus*, yeasts, and dermatophytes strains tested, and a weak activity was recorded against *Microsporum canis* and *Trichophyton rubrum* with both MLC and MIC of 400 *μ*g/mL. Izzo et al. [102] showed that among the 68 extracts tested for their antibacterial action against eight Gram-negative and Gram-positive bacteria, *C. arvensis* ethanolic extracts possessed activity against only two Gram-positive microorganisms (*Staphylococcus aureus* and *Bacillus subtilis*), while they were completely inactive on other bacteria.

The aqueous extracts of *C. arvensis* foliar tissues were examined for their activity against *F. oxysporum* f.sp. *lycopersici* isolated from tomato roots using the agar diffusion method. The toxicity of *C. arvensis* extracts against the fungus was pronounced at 4 days of incubation, with an inhibition diameter of 43.8 mm compared to the control (60 mm), reaching 75.5 mm at 16 days of incubation [103].

Likewise, the antibacterial effect of *C. arvensis* L., aerial part EO obtained by HD using a Clevenger-type apparatus was investigated against *E. coli*, *P. aeruginosa*, *Bacillus cereus*, and *S. aureus* using broth microdilution. According to the results obtained, *C. arvensis* EO (8 mg/mL) showed weak inhibitory action against *B. cereus and E. coli*. While it showed no antibacterial effect against *P. aeruginosa* and *S. aureus* [96].

The antimicrobial activities of EOs obtained by HD and MD, as well as methanolic, ether, and hexane extracts of *C. arvensis* were quantitatively studied in respective broths against nine microorganisms (*S. cerevisiae*, *C. albicans*, *M. smegmatis*, *B. cereus*, *S. aureus*, *E. faecalis*, *P. aeruginosa*, *Y. pseudotuberculosis*, and *E. coli*) using double dilution and the MIC values (*μ*g/mL) were defined. This study indicates that only the EO, obtained by HD, and the methanolic extract showed moderate effects against *B. cereus* and *S. aureus* with MIC of the order of 105–210 *μ*g, respectively. Interestingly, all extracts displayed good antituberculosis effects against *M. smegmatis* with MIC between 13.2 and 62.5 *μ*g [41]. According to Kim [104], *C. arvensis* extract had excellent antibacterial activity, and after 12 hours of culture, it inhibited the growth of *B. subtilis*, *E. coli*, and *C. albicans* by 40, 7, 35.2, and 27.5%, respectively. Moreover, the growth inhibitory effect against the positive bacteria, *B. subtilis*, was the highest.

#### 3.5.2. Antiparasitic Activity

The spectral and chemical studies on triterpenoid and sesquiterpene bioactive chemotypes of *C. arvensis* showed a significant inhibitory effect against vesicular stomatitis and rhinovirus multiplication in cell cultures, in which glycosides 1 and 2 were most effective [87, 98]. Besides, arvensoside B and arvensoside D saponins extracted from *C. arvensis* also possess an impressive impact on *Trypanosoma brucei brucei* at a MIC value of 50 and 100 *μ*g/mL, respectively [43].

*Lemna minor*, *Artimia salina* (Brine shrimps) larvae, *Callosobruchus analis*, and other parasites were tested for their susceptibility towards the methanolic extract of *C. arvensis* [99]. The insecticidal potential of *C. arvensis* pronounced a variable degree of susceptibility of strains and tested doses, in which *Callosobruchus analis* was the most sensitive insect with LD_50_ of 0.51 mg/mL. A moderate level of cytotoxicity was found with a LD_50_ value of 9.23 *μ*g/mL against brine shrimp larvae with a mortality percentage of 40, 60, and 67% at 10, 100, and 1000 *μ*g/mL, respectively [99]. Nonetheless, low toxicity (% inhibition ≤40%) at 10 and 100 *μ*g/mL and moderate effect (% inhibition = 40–50%) at 1000 *μ*g/mL were observed against *Lemna minor* [99].

#### 3.5.3. Anti-Inflammatory Activity

Some studies have shown that *C. arvensis* extracts exhibit anti-inflammatory effects. Indeed, Mascolo and collaborators showed that the injection of 100 mg/kg BW of *C. arvensis* extracts in rats induces an interesting anti-inflammatory effect by inhibiting 32% (2.68 ± 0.8) of edema formation [45]. In the same way, Abudunia et al. [44] demonstrated significant anti‐inflammatory results of *C. arvensis* extracts, in which flowers extracted by hexane solution had the best edema reduction yield of rat paw edema, with 51.08, 71.33, 63.38, and 67.33%, induced by carrageenan and experimental trauma, respectively. These outcomes indicated the contributory effects of using *C. arvensis* flower extracts in the management of inflammatory and painful conditions [44].

#### 3.5.4. Antioxidant Activities of *Calendula arvensis*

As mentioned in previous sections, *C. arvensis* is an important industrial and medicinal plant with various biological activities. To estimate, *in vitro*, the antioxidant effects of *C. arvensis* flower extracts, four different methods (DPPH, FRAP, and *β*-carotene bleaching test) were used. The methanolic, hexanolic, and organic extracts were obtained by soxhlet extraction, while the aqueous extracts were obtained by maceration. Both aqueous and methanolic extracts have free radical scavenging effects. However, the free radical scavenging effect of the methanolic extract appears to be closer to 332 mg/mL gallic acid. At all concentrations, the extracts were ranked according to their free radical scavenging potency in the following order; methanol extract > aqueous extract > hexanol extract. The EC_50_ of the methanolic and aqueous extracts are, respectively, 20.9 and 33.2 mg/mL. The reducing power using the ferric-reducing antioxidant power (FRAP) method, showed that *C. arvensis* flower extracts show significant differences in the following order; methanol extract > aqueous extract > hexanol extract. The reducing ability of *C. arvensis* methanol extract was the most potent among the three extracts (203.96 mg AAE/g Ext). Similarly, all flower extracts inhibit *β*-carotene bleaching by scavenging free radicals derived from linoleic acid. The methanol extract inhibits *β*-carotene at a rate comparable to BHT [47]. Abutaha et al. [10] showed that the 1 mg/mL of *C. arvensis* flower ethyl acetate extract (CAF EtOAC) extracted using a Soxhlet extractor present a percentage of scavenging activity (DPPH%) moderate at 50%.

In order to discover novel sources of antioxidants, aqueous and methanol/chloroform extracts of *C. arvensis* were investigated using several methods, namely, 2,2 diphenyl-1-picrylhydrazyl (DPPH) free radical scavenging assay, total antioxidant capacity (TAC), and reducing power (RP). The results showed that the antioxidant activity varies greatly not only between the extracts but also between the assays used. The reducing power values ranged from 12 Vit C equivalent mg/g DW in aqueous extracts to 100 Vit C equivalent mg/g DW in the methanol/chloroform extracts. The samples tested presented several TAC values, expressed as the number of ascorbic acid equivalents, from 19 mg vit. C equivalent/g DW in the methanol/chloroform extracts at 25 mg vit. C equivalent/g DW in aqueous extract. It was noticed that the aqueous extracts of *C. arvensis* had the highest percentage of scavenging, i.e., 48.7% and 8.7% for the methanol/chloroform extracts [38].

Belabbes et al. [88] showed that *C. arvensis* EO had the ability to reduce DPPH radical (IC_50_ = 76.2 mg/L), and more interestingly, *C. arvensis* hydrosol extract indicated the highest DPPH quenching activity (IC_50_ = 25.1 mg/L). It was found that the antioxidant activity of *C. arvensis* EO is proportional to the concentration. However, by the *β*-carotene bleaching test, the hydrosol extract (IC_50_ = 32.4 mg/L) displayed the highest potency, while the EO showed the lowest (IC_50_ = 100.1 mg/L). The BR antioxidant activity of *C. arvensis* methanolic extracts was determined as 0.024 *μ*g/mL. The activities at pH = 7.4 (TEAC method, mM Trolox eq.) show a great variability determined as 0.46 mM Trolox. By the DPPH method in methanolic solution, the results also show a great variability determined as 80.9 *μ*g/mL [31].

Ercetin et al. [49] examined the antioxidant activity of the water, methanol, ethyl acetate, acetone, dichloromethane, and n-hexane extracts of *C. arvensis* L., leaves and flowers using FRAP, ferric ion-chelating capacity, and DPPH assays at 250, 500, and 1000 *μ*g/mL. By the FRAP assay, the studied extracts had a rather weak effect, while the *C. arvensis* flower methanol extract displayed the highest absorbance determined as 0.479 at 1000 *μ*g/L. Moreover, the best scavenging activity was obtained by *C. arvensis* flower methanol extract (52.25 ± 1.34%), which was the only extract with an activity greater than 50%. Therefore, the studied extracts showed a relatively higher ferric ion-chelating activity compared to other tests. At 1000 *μ*g/L, the leaves (53.36 ± 1.23%) and flowers (53.25 ± 0.15%) of *C. arvensis* ethyl acetate extracts were found to be the best in assays.

The influence of *C. arvensis* flower methanolic and aqueous extracts at concentrations of 0.10 to 0.90 mg/mL, scavenged all types of radicals studied depending on the concentration applied. For both *C. arvensis* extracts, all the concentrations selected had an antioxidant potential lower than that of the control. At the concentration of 0.90 mg/mL, the aqueous extract scavenged approximately 60% of DPPH radicals, while at concentrations below 0.45 mg/mL no significant scavenging activity (SA) was observed compared to the control. For hydroxyl radical scavenging activity, both *C. arvensis* extracts showed high scavenging activity, while the addition of 0.45 mg/mL aqueous extract clearly scavenged the hydroxyl radical, with a scavenging activity of 34.74%. The elimination of the hydroxyl radical (SA 80–90%) was obtained with 0.9 mg/mL of the methanolic or aqueous extract. In addition, the highest antioxidant effect of *C. arvensis* aqueous extract (0.90 mg/mL) against peroxyl radical exhibited significantly lower activity (antioxidant activity, 69.28%). High concentrations of the methanolic and aqueous extracts were more effective in reducing the peroxyl radical [92].

Likewise, *C. arvensis* flowers showed effective antioxidant activity on linoleic acid and DPPH radical oxidation. This herb showed a DPPH radical scavenging activity of 409.4 *μ*g/mL as SC_50_ and an inhibitory activity of 61.1% on linoleic acid oxidation [46].

According to Messina et al. [105], six extracts of *C. arvensis* obtained by various solvents (distilled water, acetone 70%, ethanol 80%, and hexane) and by supercritical fluid extraction (SFE) showed a protective effect against superoxide anions, hydroxyl radicals, scavenging hydrogen peroxide, and oxidative damage. It was observed that the supercritical fluid extracts of *C. arvensis* presented a very remarkable antioxidant effect compared to the ethanol 80% extract (DPPH and reducing power method), in comparison to a synthetic antioxidant.

The relative levels of antioxidant activity of methanolic and aqueous extracts of *C. arvensis* of Jordanian origin were determined using the ABTS ^+^ method. There was a low variation in the total antioxidant capacity of the extracts. The total antioxidant capacity ranged from 42 to 48.4 *μ*mol TE/g DW for the methanolic and aqueous extracts, respectively [97]. Kim [104] showed that the DPPH scavenging activity of *C. arvensis* extract samples at concentrations of 10, 50, and 100 *μ*g/mL showed almost the same radical scavenging activity as the control (ascorbic acid) (88.9, 91.2, and 91.3%). Lee et al. [106] showed that with high DPPH free radical scavenging activity, the possibility of using *C. arvensis* flower extracts as a natural antioxidant was high. The DPPH free radical scavenging activity was 66.99% and 77.63% in the hot water and 80% MeOH extracts, respectively.

#### 3.5.5. Anticancer Activity

*C. arvensis* extracts have been shown to be a promising source of cytotoxic compounds. The effects of *C. arvensis* flower extracts from villages around the Rabat-Khemisset region (Morocco) were assessed on the growth of myeloid cancer cells using MTT assay and it was shown that methanol and hexane extracts (obtained by soxhlet) and aqueous extracts (obtained by maceration) exhibited important antimyeloid cancer activity (IC_50_ = 31 mg/mL). Consequently, both methanolic and aqueous extracts have been shown to be effective candidates as cytotoxic agents. Indeed, their efficacy was more pronounced compared to the hexanolic extract. At the concentration of 100 mg/mL at 24 h, it was also deduced that methanolic extract shows a maximum inhibition of 89%, testifying that this extract is a very important antimyeloid cancer agent [47].

At different concentrations, the effect of *C. arvensis* part (leaves, stems, and flowers) extracts obtained by a Soxhlet extractor using various solvents (methanol, ethyl acetate, chloroform, and hexane) against MDA-MB-231 and MCF-7 cells were estimated. Among all extracts, the flower ethyl acetate (CAF EtOAC) extract showed the highest activity, it inhibited cell growth in a concentration-dependent manner. This extract displayed IC_50_ values of 70 and 78 *μ*g/mL in MCF-7 and MDA MB-231 cells, respectively. However, the flower hexane extract presented a moderate cytotoxic effect, but the methanol and chloroform extracts showed no activity [10].

The study of Attard and Cuschieri [48] indicated that the petroleum ether extract of *C. arvensis* aerial part is relatively nontoxic to peripheral lymphocytes, suggesting its use as an immune stimulant. In fact, all the extracts studied presented a concentration-dependent effect. Phytohemagglutinin (PHA) (SC_50_ < 0.001 *μ*g/mL) and the petroleum ether extract (SC_50_ 0.089 *μ*g/mL) of *C. arvensis* obtained by maceration demonstrated an increase in proliferation compared to the other extracts (IC_50_ > 10 *μ*g/mL). However, the most active extract of *C. arvensis* was the petroleum ether extract. In contrast, it has been reported that *C. arvensis* extract is a safe natural product, as at least it did not induce any cytotoxicity at 100 *μ*g/mL. The cytotoxicity of this extract was 0, 0.1, 0.3, 15.1, and 28.0% at increasing concentrations of 10, 100, 250, and 500 *μ*g/mL, respectively [104].

Quetin-Leclercq et al. [95], showed that the saponins isolated from *C. arvensis* are very active against certain cancer cells (human HeLa tumor cells, Flow 2002 noncancer human cells, mouse 313 noncancer fibroblasts, and mouse B16 melanoma cells) as much as the reference compound, strychnopentamine. At concentrations of 10 *μ*g/mL and above, they exhibited some degree of cytotoxicity, while the concentration of 50 *μ*g/mL was the most potent.

According to Ullah et al. [107], the toxic potential of the methanol crude extract of *C. arvensis* whole plant was evaluated by a *Lemna minor* bioassay. It was observed that *C. arvensis* exhibits dose-dependent toxicity towards *Lemna minor*, with low toxicity (% inhibition ≤40%) at 10 and 100 *μ*g/mL, and moderate activity (% inhibition = 40–50%) at 1000 *μ*g/mL. A moderate level of cytotoxicity was found to have an LD_50_ value of 9.23 *μ*g/mL for saltwater shrimp larvae. Zihlif et al. [108] found that the *C. arvensis* ethanol extract has a potential antiangiogenic activity. A selectivity was demonstrated against the proliferation of endothelial cells, indicating a direct inhibitory power on the tumor angiogenesis key step. The activity of this extract shows more than 50% growth inhibition. After 72 h at 50 *μ*g/mL, an important antiproliferative activity was also shown against the MCF7 cell line. The extract reduced the growth of these cells to less than 30%. While it showed weak antiangiogenic activity on PFL cell proliferation at 50 *μ*g/mL. Interestingly, it showed potent activity against HUVEC cell proliferation with IC_50_ = 28.7 *μ*g/mL.

#### 3.5.6. Enzyme Inhibitory Activity

The investigation of the enzymatic inhibitory effects in different solutions (distilled water, methanol, ethyl acetate, acetone, dichloromethane, and n-hexane) of *C. arvensis* leaf and flower extracts, collected from the vicinity of Bodrum town in the province of Mugla (Turkey), against butyrylcholinesterase (BChE) and acetylcholinesterase (AChE) was performed by Ercetin et al. [49]. Their outcomes highlighted the high efficiency of *C. arvensis* flower ethyl acetate extract in AChE inhibition assay (31.24 ± 1.29%) compared to *C. officinalis* extracts. This effect has been attributed to the presence of compounds derived from flavonoids and triterpenes (tannins, malic and salicylic acid, mucilages, etc.) found in the flower methanolic extract of this specie [49].

#### 3.5.7. Antimutagenic Activity

Elias and Meo [89] used the microsuspension technique of the Salmonella/microsomal assay (Ames test) to investigate the antimutagenic potential of natural saponins isolated from the dried aerial parts of *C. arvensis*. Their preliminary findings demonstrated that the saponins were not toxic for the tested strains of *Salmonella typhimurium* (TA 100, TA 98, and TA 97) for doses up to 400 *μ*g. The use of benzo[a]pyrene (BaP) and a mutagenic urine concentrate from a healthy smoker (consumed 40 cigarettes per day), showed that the four products (D, C, B, and A) of the studied plant exert an antimutagenic effect against 1 *μ*g of BaP and 5 *μ*L of smoker's urine [89]. Probable antimutagenic mechanisms of saponins have been reported to be the modification of cell surface activity and cell membrane structure [89]. In their research paper, a group of Australian chemists concluded that the saponin arvensoside B sugar units (chemically named 1⟶3-Galactopyranosyl-D-glucopyranosyl-3-O-oleanolic acid) strongly influence the hemolytic activity of oleanolic acid disaccharides [100]. Not only that but also the linkage positions 3 or 4 either arvensoside B or its analog, calenduloside found in *C. officinalis* are structural requirements for high potency [100].

#### 3.5.8. Immunomodulatory Activity

The immunomodulatory activity of various Maltese plant extracts (i.e., *Carlina involucrata* Poir, *Galactites tomentosa* Moench, *Leontodon tuberosus* L, *Glebionis coronaria* L. Tzvelev, *Aster squamatus* (Sprengel) Hieron, *Reichardia picroides* L. Roth, *Sonchus oleraceus* L, *Calendula arvensis* L, *Inula crithmoides* L, and *Dittrichia viscosa* L. Greuter) of the Asteraceae family was performed on human peripheral T-lymphocytes*in vitro* using macerated dried aerial parts in different solvents [48]. The authors observed marked effects in lymphocytes treated with extracts, but only *C. arvensis* petroleum ether extract was able to activate the lymphocytes and pronounce blastogenesis similar to that of PHA, which can be used as a potential immune booster [48].

#### 3.5.9. Wound Healing Activity

The local application of a mixture of *H*. *perforatum* and *C*. *arvensis* EOs in the epithelial reconstruction of surgical wounds in childbirth with cesarean section in Italy on a group of 24 patients induced a wound size reduction of 37.6 ± 9.9% versus a reduction of 15.83 ± 4.64% in the control group [101]. The above-mentioned results are very encouraging to shed more light on *C. arvensis* for further investigations on the extraction methods according to the different plant parts, isolation of the main biomolecules, and the elucidation of their mechanism of action.

## 4. Conclusion and Perspectives

In this paper, the sources and main pharmacological characteristics of *C. arvensis* have been investigated and presented. The analysis of several published studies revealed that this natural compound has significant biological properties, especially its wide range of use in folk medicine by several ethnic groups around the world as a poultice, antidiabetic, antispasmodic, antiemetic, antihelmintic, antirheumatic, diaphoretic, and also as a treatment against neurological disorders, microbial disease, ENT and typhoid diseases, skin diseases, digestive disorders, and high blood pressure. Additionally, all *C. arvensis* flower extracts exhibit an inhibitory effect against most fungi and bacteria species. In addition, due to the phytochemical constituents of *C. arvensis*, including polyphenols, flavonoids, terpenoids, tannins, and alkaloids, and also from its antimicrobial, antioxidant, and anti-inflammatory properties, this medicinal plant can be considered an interesting bioactive compound to explore different molecular and cellular pathways in cancer prevention. However, a greater comprehension of its pharmacokinetics and pharmacodynamics is warranted for its incorporation as a drug in cancer therapy and other pathologies. In this context, the assessment of its safety potential is also required, through a more in-depth toxicological survey.

## Figures and Tables

**Table 1:** Ethnomedicinal use of *C. arvensis*.

Area of study	Use part	Mode of preparation	Traditional use	References
The Khattak tribe of Chonthra Karak (Pakistan)	Flowers	Powder	Strengthen eyesight, treat heart disease and heal wounds	[52]
Upper Dir, Khyber Pakhtunkhwa (Pakistan)	Root and leaves	Poultice	Treat eye disorders, oral sore, and inflammation	[53]
The tribal population of the north-west Frontier Province (Pakistan)	Leaves	Crushed	Wound healing	[54]
District Attock (Pakistan)	Flowers	—	Strengthen eyesight, treat heart disease, and promote skin healing	[55]
Madyan Valley in district Swat (Pakistan)	Leaves and flowers	—	Used as an anthelmintic, tonic, and diaphoretic	[56]
Jatlan Azad Jammu, Kashmir (Pakistan)	—	Decoction	Used as a diaphoretic and antiemetic. Treat digestive ailments	[57]
Karak, Khyber Pakhtunkhwa (Pakistan)	Leaves and flowers	—	Treat toothache	[58]
Malam Jabba Valley of Swat (Pakistan)	Leaves and flowers	—	Used as a tonic, diaphoretic, and anthelmintic	[37]
The Alaşehir and its surrounding (Manisa/Turkey)	Aerial parts	Infusion, mash, and medical oil	Treated skin cancer. Used in burn care	[32, 33]
Erzincan (Turkey)	Flowers	Decoction	Used as a hepatoprotector, germicide, and fungicide	[34]
Yaslıca Town and Arıkök neighborhood in the Bozova district of Şanlıurfa province (Turkey)	Flowers	Decoction	Fungal treatment	[24]
Targuist area (North of Morocco)	Flowers	Cataplasm	Used as an anti-inflammatory and antiseptic	[14]
The Tiaret Mountains (Western Algeria)	—	—	Used as a depurative, emmenagogue, antispasmodic, and stimulant of hepatic activity and bile secretion	[59]
The Mountains Tessala (Western Algeria)	Flowers	Infusion	Used as a depurative, emmenagogue, antispasmodic, and stimulant of hepatic activity and bile secretion	[60]
	Leaves	Maceration	Calm vomiting and internal ulcers Erase warts, calluses body, fistula, frostbite, and skin lesions	
The Moroccan Rif	Flowers	Infusion	Treat diabetes	[16]
Aguelmouss (Khenifra, Morocco)	—	—	Wound healing	[26]
Sardinia (Italy)	Leaves	Teas or decoction	Used as an emmenagogue, diaphoretic, diuretic, sedative, anti-inflammatory	[31]
Aguelmouss, Khenifra Province (Morocco)	—	—	Treated neurological, microbial affections, ENT, and typhoid diseases	[17]
The Canary islands (Spanish)	Aerial parts	—	Used as an emmenagogue and aperitive	[27]
Rabat-Sale-Kenitra Regions (Morocco)	Leaves and flowers	Decoction	Treat diabetes	[15]
The Mt. Pelion Area (Greece)	Leaves, stems, and flowers	—	Treat acne and diaper rash	[61]
The Mainarde Mountains (Central-Southern Apennine, Italy)	—	Macerated and ointment	Treat burns, sunburn, chilblains, joint pain, and stings	[62]
Western Gironès (Catalonia, Iberian Peninsula)	Aerial parts	Bath	Ocular antiseptic	[29]
The mountains of Tlemcen (Western Algeria)	—	—	Disinfectant and antihemorrhagic Treat influenza, transpiration, and hemostatic action	[63]
District Swat (Pakistan)	Leaves and flowers	—	Used as scrofula, diaphoretic, antihelminthic, and tonic	[64]
Swat Valley (Pakistan)	Leaves and flowers	Powder	Used as scrofula, tonic, diaphoretic, anthelmintic Treat mucus discharge	[65, 66]
Swat (Pakistan)	Flower and leaves	Shoot	Used as stimulant, antispasmodic, and astringent. Treat wounds and injuries	[67]
Gujranwala Region, Punjab (Pakistan)	Leaves and flowers	Extract	Severe pains	[68]
Oulad Daoud Zkhanine (Nador Province, Morocco)	Whole plant	Decoction	Treat digestive disorders. Used for hair care	[19]
Kohistan Valley, Khyber Pakhtoonkhwa (Pakistan)	Flowers	—	Strengthen eyesight. Treated heart and skin diseases	[69]
Thz M'Zab valley (Algeria)	Flowers	Infusion	Treat high blood pressure	[20]
Sawans Valley Mianwali, Punjab (Pakistan)	—	—	Wound healing	[70]
The Talash Valley of Dir Lower (Pakistan)	Flowers	Juice	Treat toothache	[71]
Syria's western region (Tartus and Latakia)	Aerial parts	Infusion	Treat varicose veins, sore eyes, wounds, sprains, bites and stings, and skin problems	[72]
The villages of Lotzorai and Escolca (Sardinia, Italy)	Flowers	Infusion and cataplasm	Used as an antispasmodic Treat burns	[22]
The Taza region (Morocco)	Stems and flowers	Decoction	Treat diabetes	[25]
Tehsil Razzar District Swabi (Pakistan)	Leaves and flowers	Decoction in oil	Treat rheumatic diseases	[21]
Calabria Region (Southern Italy)	Flower heads	Decoction	Treat wounds, contusions, and burns	[30]
Muzaffarabad, Azad Jammu, and Kashmir (Pakistan)	Leaves and flowers	—	Used as astringent, expectorant, and antispasmodic. Treat varicose veins	[35]
Bosnia and Herzegovina	Flowers	Infusion and decoction	Treat lung cancer and treat liver and skin diseases	[73]
The Ripollès district (Spain)	Inflorescences	Liniment	Used as antiecchymotic	[28]
Alaşehir (Manisan Turkey)	Aerial parts	Infusion, maceration, and oil	Treat skin cancer and treat burns	[33]
Sarigöl district (Manisa, Turkey)	Aerial parts	Infusion, mash, and lotion	Treat sores, burns, skin cancers, and athletes' feet	[32]
The Bissa region (Northeastern Dahra Mountains, Algeria)	Leaves and roots	Decoction and infusion	Treat rheumatism	[74]
Malam Jabba, Swat (Pakistan)	Leaves and flowers	—	Used as a tonic, diaphoretic, and anthelmintic	[18]
Chagharzai Valley, District Buner (Pakistan)	Leaves and shoots	—	Treat skin diseases	[75]
Margalla Hills National Park, Islamabad (Pakistan)	Leaves and flowers	—	Used as an antispasmodic, emmenagogue, and wound healing	[36]
Azad Jammu And Kashmir state (Pakistan)	All parts	Decoction	Treat diaphoretic and antiemetic disorders	[23]
Mihalgazi (Eskişehir, Turkey)	Leaves and capitulum	Poultice	Treat skin diseases	[76]
South-West of Pakistan	Leaves and stem	Infusion	Treat diabetes	[77]

**Table 2:** Chemical compounds of *C. arvensis*.

Countries	Parts	Extracts/EOs	Compounds groups	Compounds	References
Italy	Aerial parts	Methanolic extract	Saponins	3-O-(*β*-D-galactopyranosyl-(1⟶3)-*β*-D-glucopyranosyl) oleanolic acid-28-O- *β*-D-glucopyranoside, 3*β*-O-(*β*-D-galactopyranosyl-(1⟶3)-*β*-D-glucopyranosyl) oleanolic acid, 3*β*-O-(*β*-D-galactopyranosyl-(1⟶3)-*β*-D-glucopyranosyluronic acid) oleanolic acid-28-O- *β*-D-glucopyranoside, 3*β*-O-(*β*-D-galactopyranosyl-(1⟶3)-*β*-D-glucopyranosyluronic acid) oleanolic acid	[84]
Italy	Aerial parts	Methanolic extract	Saponins	4-O-(*β*-D-fucopyranosyl)-4-alloaromadendrole, and three derivatives of arvensoside B	[84]
Tunisia	Aerial parts	Methanolic extract	Saponins	Arvensoside A and arvensoside B	[85]
Tunisia	Aerial parts	Ethanolic extract	Saponins	Calenduloside D, calenduloside C, arvensoside B, and arvensoside A	[86]
Italy	Aerial parts	Chloroform extract	Saponins	3-O-(*β*-D-galactopyranosyl-(1⟶3)-*β*-D-glucopyranosyl) oleanolic acid-28-O- *β*-D-glucopyranoside, 3*β*-O-(*β*-D-galactopyranosyl-(1⟶3)-*β*-D-glucopyranosyl) oleanolic acid, 3*β*-O-(*β*-D-galactopyranosyl-(1⟶3)-*β*-D-glucopyranosyluronic acid) oleanolic acid-28-O- *β*-D-glucopyranoside, 3*β*-O-(*β*-D-galactopyranosyl-(1⟶3)-*β*-D-glucopyranosyluronic acid) oleanolic acid, 4-O-(*β*-D-fucopyranosyl)-4-alloaromadendrole, 4-O-(*β* -D-fucopyranosyl)-4-alloaromadendrol-2″-methylpropanoyl esters, 4-O-(*β* -D-fucopyranosyl)-4-alloaromadendrol -2″-methyl-2″-butenoyl esters	[87]
Italy	Aerial parts	Methanol- diethyl ether-petroleum ether (1 : 1 : 1)	Sesquiterpeneglycosides	3*α*,7*β*-dihydroxy-5*β*,6*β*-epoxyeudesm-4(15)-ene-11-(O-*β*-D-fucopyranoside-2′,4′ -diangelate-3′-acetate), 7*β*-Hydroxy-3*β*-acetoxy-5*β*,6*β*-epoxyeudesm-5(15)-ene-11-(O-*β*-D-ficopyranoside-2′,4′-diangelate-3′-acetate), 3*α*,7*β*-Dihydroxy-5*β*,6*β*-epoxyeudesm-4(15)-ene-11-(O-*β*-D-fucopyranoside-2′,4′-diangelate-3′-isobutyrate), 3*α*,7*β*-dihydroxy -5*β*, 6*β*-epoxyeudesm-4(15)-ene-11-(O-*β*-D-fucopyranoside-2′, 4′-diangelate-3′-methylbutyrate), and 3*α*,7*β*-dihydroxy-15-acetoxyeudesm-4(5)-ene-11-(O-*β*-D-fucopyranoside-2′,4′-diangelate-3′-acetate)	[12]
Morocco	Flowers	Aqueous extract	Phenolic and flavonoid contents	Total phenolic contents: 47.89 ± 2.34 mg GAE/g DW Total flavonoid contents: 74.93 ± 1.50 mg RE/g DW	[47]
		Hexanolic extract		Total phenolic contents: n.d Total flavonoid contents: n.d	
		Methanolic extract		Total phenolic contents: 50.26 ± 0.18 mg GAE/g DW Total flavonoid contents: 174.93 ± 5.21 mg RE/g DW	
Saudi Arabia	Flowers	Ethyl acetate extract	Fatty acids	Stearic acid, oleic acid, linoleic acid, linolenic acid, palmitic acid, and palmitoleic acid	[10]
			Phenolic and flavonoid contents	Total phenolic contents: 354 *μ*g/mL Total flavonoid contents: 270 *μ*g/mL	
Pakistan	Leaves	Methanolic/chloroform extract	Phenolic and flavonoid contents	Terpenoids, tannins, saponins, and alkaloids Total phenolic contents: 25 ± 2.0 mg GAE/g DW Total flavonoid contents: 8.0 ± 2.3 mg QE/g DW	[38]
		Aqueous extract	Phenolic and flavonoid contents	Total phenolic contents: 24.6 ± 3.5 mg GAE/g DW Total flavonoid contents: 10.2 ± 2.5 mg QE/g DW	
Malta	Aerial parts	Petroleum ether extract	Phytochemical screening	Flavonoids	[48]
		Chloroform extract		Alkaloids, proteins, and terpenoids	
		Ethanolic extract		Alkaloids, flavonoids, and proteins	
		Aqueous-ethanolic extract (1 : 1)		Flavonoids, proteins, and terpenoids	
		Distilled water		n.d	
Algeria	Aerial parts	Essential oil	Terpenoids	*γ*-terpinene, limonene, *p*-cymene, 2-pentylfurane, myrcene, *β*-pinene, sabinene, *α*-pinene, *α*-thujene, and (Z)-hex-3-en-1-ol, (E)-sabinene hydrate, nonanal, terpinen-4-ol, 7-*β*-silphiperfol-5-ene, *α*-longipinene, *α*-copaene, (E)-*β*-caryophyllene, *β*-acoradiene, (E)-*β*-farnesene, *α*-himachalene, allooromadendrene, *γ*-muurolene, *γ*-curcumene, germacrene D, zingiberene, bicyclogermacrene, (E,E)-*α*-farnesene, *γ*-cadinene, *β*-curcumene, *β*-sesquiphellandrene, (E)-*α*-bisabolene, epi-globulol, globulol, guaiol, viridiflorol, zingiberenol 1 and 2, eremoligenol, *τ*-cardinol, *τ*-muurolol, *α*-cadinol, (Z,Z)-farnesol, *α*-bisabolol, (E,Z)-farnesol, heptadecane, *α*-oxo-bisabolene, nonadecane, eicosane, heneicosane, (E)-phytol, tricosane, tetracosane, and pentacosane	[88]
		Hydrosol extract	Terpenoids	Terpinen-4-ol, globulol, guaiol, zingiberenol 1, zingiberenol 2, eremoligenol, *τ*-cardinol, *τ*-muurolol, *α*-cadinol, *α*-bisabolol, (E,Z)-farnesol, and *α*-oxo-bisabolene	[88]
Italy	Aerial parts	Steam distillation extract	Aldehydes, esters, sulfur compounds, alkyl pyrazines, ketones, lactones, and terpenoid compounds	Ethyl butyrate, 2-methyl-3-furanthiol, methional, 1-octen-3-one, ethyl hexanoate, 2-6-Dimethyl-3 ethyl pyrazine, (E)-2-nonenal, (E,E)-2,4-octadienal, 5-methyl-2-furanaldehyde, citronellol, phenethylacetate, *α*-terpineol, lactone-like, and *δ*-decalactone	[11]
Italy	Aerial parts	Methanolicextract	Saponins	3-O-(*β*-D-galactopyranosyl-(1⟶3)-*β*-D-Glucopyranosyl) oleanolic acid-28-O- *β*-D-Glucopyranoside, 3*β*-O-(*β*-D-galactopyranosyl-(1⟶3)-*β*-D-glucopyranosyl) oleanolic acid, 3*β*-O-(*β*-D-galactopyranosyl-(1⟶3)-*β*-D-glucopyranosyluronic acid) oleanolic acid-28-O-*β*-D-glucopyranoside, 3*β*-O-[*β*-D-galactopyranosyl-(1⟶3)-*β*-D-glucopyranosyluronic acid) oleanolic acid, 3-O-{*β*-D-ga1actopyranosyl-(1⟶3)(*β*-D-glucopyranosyl-(1⟶4))-*β*-D-glucopyranosyl}oleanolic acid (28⟶1)-*β*-D-glucopyranosyl ester, 3-O-{*β*-D-ga1actopyranosyl-(1⟶3)(*β*-D-glucopyranosyl-(1⟶4))-*β*-D-glucopyranosyl}oleanolic acid	[87]
France	Aerial parts	Hydro-ethanolic extract (60%)	Saponins	3-O-(*β*-D-galactopyranosyl-(1⟶3)-*β*-D-glucopyranosyl) oleanolic acid-28-O- *β*-D-glucopyranoside, 3*β*-O-(*β*-D-galactopyranosyl-(1⟶3)-*β*-D-glucopyranosyl) oleanolic acid, and 3-O-(*β*-D-glucopyranosyl-(1⟶2)-*β*-D-galactopyranosyl-(1⟶3)-*β*-D-glucopyranosyl) oleanolic acid-28-O- *β*-D-glucopyranoside, 3-O-(*β*-D-glucopyranosyl-(1⟶2)- *β*-D-galactopyranosyl-(1⟶3)-*β*-D-glucopyranosyl) oleanolic acid	[89]
Turkey	Flower parts	Methanolic extract	Phenolic and flavonoid contents	Total phenoliccontents: 118.18 ± 10.29 mg GAE/g extract Total flavonoid contents: 74.14 ± 3.09 mg QE/g extract	[49]
Portugal	Aerial parts	Hexane extract	Carboxylic acids and derivatives	Stearic acid, *α*-linolenic acid, linoleic acid, palmitic acid, quinic acid, citric acid, and tetracosanoic acid	[90]
			Carbohydrates	L-threonic acid, D-(−)-tagatofuranose, D-(−)-fructofuranose, D-(−)-fructopyranose, D-(−)-psicopyranose, D-(+)-mannopyranose, D-(+)-galactopyranose, *β*-D-glucopyranose, D-gluconic acid, galactaric acid, sucrose, and cellobiose	
			Terpenoids	Neophytadiene, phytol, *α*-bisabolol, 8,14-cedranoxide, stigmasterol, stigmast-5-ene, amyrin, lup-20(29)-en-28-al, and 3-oxo-ursan-28-oic acid	
			Alcohols, alkanes, amino acids, and pyrrolizidine Alkaloids	Myo-inositol, 1H-benzocyclohepten-9-ol, 1-hexacosanol, untriacontane, 4-aminobutanoic acid, and an isomer of platynecine derivative	
Portugal	Aerial parts	Methanolic extract	Phenolic compounds	Isomeric form hydroxy ferulic acid hexoside, 5-O-caffeoylquinic acid, 4-O-caffeoylquinc acid, caffeic acid, sinapic acid hexoside, sinapic acid, hexoside derivative, caffeoylshikimic acid, 3,4-O-dicaffeoylquinic acid, 5-O-feruloylquinic acid, quercetin hydrate, apigenin-8-C-pentose-6-chexose or apigenin-8-chexose-6-C- pentose, quercetin dihexoside, quercetin-3-O-rutinoside, quercetin-3-oneohesperidoside, quercetin-3-omalonylhexoside, isorhamnetin-3-O-hexoside, quercetin acetyl hexoside, quercetin hexoside I, apigenin-O-hexosylpentosyl, protocatechuic acidpentoside, quinic acid with an aldonic residue, ligstroside hexoside, calendasaponin A, and calenduloside G isomer	[42]
			Alcohols, aldehydes, aliphatic hydrocarbons, esters, and ketones	(Z)-3-Hexen-1-ol, hexanal, (E)-2-hexenal, decanal, nonanal, dodecane, ethyl hexanoate, ethyl octanoate, and thymol methyl ether	
Portugal	Flower parts	Fresh petals	Alcohols, aldehydes, aliphatic hydrocarbons, esters, ketones, sesquiterpenes, and terpenes	Aromadendrene, (E)-*α*-bergamotene, *α*-caryophyllene, *α*-gurjunene, *β*-cubebene, *β*-copaene, *β*-bourbonene, *α*-copaene, *α*-ylangene, *α*-cubebene, and *δ*-elemene, *β*-caryophyllene, *γ*-muurolene, alloromadendrene, germacrene D, *α*-muurolene, *δ*-cadinene, *α*-cadinene, *α*-calacorene, epi-*α*-cadinol, *α*-thujene, styrene, limonene, *α*-terpinene, *α*-phellandrene, *β*-myrecene, sabinene, and *α*-pinene, (E)-*β*-ocimene, (Z)-sabinene hydrate, *p*-cymenene, linalool, *δ*-terpinene, *β*-thujone, perillene, alloocimene, neo-allo-ocimene, 3-thujen-2-one, terpin-4-ol, terpinolene, estragole, (Z)-cadina-1(6),4-diene, and (E)-cadina-1,4-diene	[91]
		Water: acetone (6 : 4; v/v) extract	Monomeric anthocyanins, total flavonoids, and hydrolysabletannins contents	Total flavonoids: 0.35 ± 0.10 mg QE/g DW hydrolyzable tannins: 3.7 ± 1.8 mg TAE/g DW Total monomericanthocyanins: 0.07 ± 0.01 mg Cy 3-glu/gDW	
Serbia	Flower parts	Methanolic extract	Phenolic and flavonoid contents	Total phenolic contents: 15.12 ± 0.40 mg CAE/g DW Total flavonoid contents: 5.31 ± 0.36 mg RE/g DW	[92]
		Water extract	Phenolic and flavonoid contents	Total phenolic contents: 14.49 ± 0.38 mg CAE/g DW Total flavonoid contents: 5.26 ± 0.36 mg RE/g DW	
Turkey	Aerial parts	Butanolic extract from methanolic extract (liq-liq)	Saponins	Arvensoside C, arvensoside A, arvensoside B, glycoside C, and calenduloside D	[93]
		Ethyl acetate extract from methanolic extract (liq-liq)	Flavonol glycosides	Quercetin 3-O-*β*-D-galactopyranoside, quercetin 3-O-*β*-D-glucopyranoside, and isorhamnetin 3-O-*β*-D-glucopyranoside	
Portugal	Stems and leaves	Chloroform extract	Phytosterols	*β*-sitosterol	[94]
France	Aerial parts	Essential oil	Terpenoids	(Z)-Hex-3-en-1-ol, *α*-thujene, *α*-pinene, sabinene, *p*-cymene, myrcene, *β*-pinene, *β*-phellandrene, limonene, *p*-cymenene, linalool, terpinolene, *cis*-Sabinene hydrate, 4-methyl acetophenone, terpinen-4-ol, dill ether, *cis*-piperitol, decanal, *α*-terpinen-7-al, *p*-menth-1-en-9-ol, *α*-cubebene, (E,E)-2,4-decadienal, geranyl acetate, *α*-copaene, iso-ledene, aromadendrene, trans-a-bergamotene, (E)-*β*-caryophyllene, *α*-gurjunene, *β*-cubebene, *β*-bourbonene, *cis*-muurola-3,5-dienev *α*-humulene, allo-aromadendrene, carota-5,8-diene, *γ*-muurolene, *α*-curcumene, *γ*-humulene, germacrene-D, trans-muurola-4(14),5-diene, eremophila-1(10),7-diene, 4-epi-cubebol, bicyclogermacrene, ledene, valencene, *α*-muurolene, *β*-bisabolene, *γ*-cadinene, cubebol, calamenene (*cis* and/or *trans*), presilphiperfolane-9a-ol, *δ*-cadinene, zonarene, cadina-1,4-diene, *α*-calacorene, *α*-cadinene, *α*-agarofurane, (E)-nerolidol, *β*-calacorene, cis-sesquisabinene hydrate, palustrol, spathulenol, germacrene D-4-ol, caryophyllene oxide, gleenol, globulol, cubeban-11-ol, germacradien-11-ol, *β*-oplopenone, guaiol, ledol, zingiberenol, *α*-cadinol, *τ*-cadinol, *τ*-muurolol, cubenol, cadin-4-en-7-ol, 1-epi-cubenol, 10-epi-*γ*-eudesmol, 1,10-di-epi-cubenol, (Z,Z)-farnesol, *α*-bisabolol, (E,Z)-farnesol, cedryl methyl ketone, and (E)-phytol	[7]
France	—	—	Monodesmosidesaponins	Arvensoside B and arvensoside D	[95]
Turkey	Aerial parts	Essential oil	Terpenoids	*α*-pinene, *α*-cubebene, *α*-longifolene, *α*-copaene, *α*-gurjenene, *β*-cubebene, *β*-caryophyllene, epi-zonarene, *α*-caryophyllene, ledene, bicyclosesquiphallendrene, germacrene D, *α*-muurolene, *δ*-cadinene, *β*-sesquiphallendrene, cadina-1,4-diene, calamenene, epi-cubebol, neophytodieneisomer, palustrol, cubebol, ledol, cubenol, 1-epi-cubenol, 1,10-di-epi-cubenol, *β*-oplopenene, viridiflorol, sesquisabinene hydrate, hexahydrofarnesyl acetone, spathulenol, zingiberenol, *τ*-cadinol, *δ*-cadinol, *α*-bisabolol, *α*-cadinol, and phytol	[96]
Jordan	Aerial parts	Aqueous extract	Phenolic content	Total phenolic contents: 16.8 ± 0.7 mg GAE/g DW	[97]
		Methanolic extract		Total phenolic contents: 12.3 ± 0.4 mg GAE/g DW	
France	—	—	Saponins	Arvensoside B and arvensoside D	[43]
Turkey	Fresh plant	Essential oil	Terpenoids, hydrocarbons, and other compounds	*α*-thujene, *γ*-terpinene, (Z)-*β*-ocimene, *p*-mentha-1(7),8-diene, limonene, *α*-phellandrene, myrcene, *β*-pinene, sabinene, *α*-pinene, *p*-cymenene, neo-allo-ocimene, terpinen-4-ol, anethofuran, *α*-terpinen-7-al, 7-epi-silphiperfol-5-ene, iso-ledene, *α*-gurjunene, *β*-elemene, *β*-bourbonene, *α*-copaene, E-caryophyllene, *β*-gurjunene, germacrene D, *α*-guaiene, (E)-*β*-farnesene, *α*-humulene, *β*-selinene, viridiflorene, *α*-selinene, 2-norpinene, *δ*-amorphene, *α*-cuprenene, *α*-muurolol, epi-*α*-muurolol, *α*-acerenol, (Z)-sesquilavandulol, viridiflorol, *α*-agorofuran, ledol, E-phytol, (Z)-*α*-santalol, 2E-3,7,11,15-tetramethyl-2-hexadecen-1-ol, Z-Jasmone, hexahydrofarnesyl acetone, eicosane, heneicosane, docosane, tricosane, tetracosane, pentacosane, benzaldehyde, nonanal, methyl salicylate, decanal, and ethyl linoleoate	[41]

n.d.:not detected; mg Cy 3-glu/g DW: mg cyanidin-3-glucoside/g DW; mg QE/g DW: mg of quercetin equivalent/g DW; mg TAE/g DW: mg of tannic acid equivalent/g DW; mg GAE/g DW: mg gallic acid equivalent/g DW; mg RE/g DW: rutin equivalent; mg QE/g DW: quercetin equivalent/g DW; mg CAE/g DW: chlorogenic acid equivalents/g DW.

## Data Availability

The data used to support the findings of this study are included in the article.

## Abbreviations

*μ*mol TE/g DW: *μ*mol of Trolox equivalent per gram of dry weight
ABTS: 2,2′-azino-bis(3-ethylbenzothiazoline-6-sulfonic acid)
BHT: Butylohydroxytoluene
DPPH: 2,2 Diphenyl-1-picrylhydrazyl
EC50: Median effective concentration
ENT: Ear, nose, and throat
FAB: Fast atom bombardment
FRAP: Ferric-reducing antioxidant power
GC/MS: Gas chromatography/mass spectrometry
GC-FID: Gas chromatography coupled with flame-ionization detection
HD: Hydrodistillation
LD50: Median lethal dose
MD: Microwave distillation
mg CAE/g DW: mg of chlorogenic acid equivalent per gram of dry weight
mg GAE/g DW: mg of gallic acid equivalent per gram of dry weight
mg QE/g DW: mg of quercetin equivalent per gram of dry weight
mg RE/g DW: mg of rutin equivalent per gram of dry weight
mg TAE/g DW: mg of tannic acid equivalent per gram of dry weight
MIC: Minimum inhibitory concentration
MIKE: Mass-analyzed ion kinetic energy
MLC: Minimal lethal concentration
MTT: 3-(4,5-dimethylthiazol-2-yl)-2,5-diphenyltetrazolium bromide
NMR: Nuclear magnetic resonance
PHA: Phytohemagglutinin
RP: Reducing power
SA: Scavenging activity
SC50: Median stimulatory concentration
SFE: Supercritical fluid extraction
TAC: Total antioxidant capacity
TEAC: Trolox equivalent antioxidant capacity
UPLC–MS: Ultraperformance liquid chromatography–MS.
